# Progress in Surgery

**Published:** 1896-09-19

**Authors:** 


### Progress in Surgery.

### GENERAL SURGERY.

**Antiseptics.**—It is refreshing to find a stand being made against the craze for new antiseptics. Ahlfeld and Vahle[1] report their discouraging experiments concerning the efficiency of quinosol as an antiseptic. Even a solution of the strength of 3 per cent. they have found not altogether to be relied on. Moreover, they state that it is not so innocent as Kossmann supposed it was. Dr. E. Witte, of Berlin, also treats of quinosol, and makes some wholesome remarks about the quest for new antiseptics. In corrosive sublimate, in carbolic acid, and in lysol, he says we have tried antiseptics; years of observation have taught us the bright and the shady side of their action. The case for quinosol has by no means been made out. Dr. Witte's experience has been contrary to that of Kossmann and Ostermann, who state that it is non-irritating to wounds. In two instances he applied quinosol in substance to the cavities left after the removal of glands, and each time such intense burning pain set in that the patient begged to have it taken out. Although he has not observed symptoms of poisoning, he insists that we cannot be sure they will not occur. A minor objection to quinosol is that it stains the skin and instruments, although the stain can be removed without much trouble. Quinosol is particularly unsuitable for vaginal irrigation, for it is highly astringent. MM. A. Trillat, Fayollat, and Foley[2] have added (the two latter in their inaugural Thèses, Lyon, 1895[3]) some valuable information respecting disinfection by means of vapours of formol (formic aldehyde) with regard to its practical value for apartments and for a great many other objects. The experiments of MM. Fayollat and Foley prove that the poisonous qualities of formol are very feeble compared to its germicidal properties. Walter's[4] series of experiments pronounce in favour of formalin as an antiseptic. It can be used in the solid, liquid, or gaseous forms. A solution of 1 in 10,000 prevents the growth of the bacilli of anthrax, cholera, typhoid, diphtheria, and of the staphylococcus pyogenes aureus. A 1 per cent. solution in thirty minutes kills pure cultures of almost all forms of pathogenic bacteria. A concentrated solution of formic aldehyde acts as a caustic, destroying diseased tissues. Schleich[5] gives details of wound treatment by means of formalin-gelatine in powder. The powder is prepared by pouring twenty-five drops of Schering's pure formalin solution over 500 g. of dissolved gelatine, and then drying over formalin vapour. As it can be bent or moulded when heated into any required form, and as it becomes replaced by fibrous tissue in the body, the author suggests its use in plastic surgery for filling up all kinds of defects. Gottstein[6] found experimentally that impregnated with lime salts and moulded between the ends of a resected bone, a firm calcified tissue continuous with the true osseous tissue takes its place, thus producing artificial bone. The latest and cheapest disinfecting substance known is that recently described by Dr. S. T. Bartochewitch[7], and derived from petroleum. Petroleum refuse is treated with strong sulphuric acid, and afterwards with a solution of caustic potash. A yellowish-brown mass is obtained, from which emulsions of variable consistency may be prepared. Comparing the result of its disinfecting action upon fæcal matter with that of quicklime or other products used for this purpose, the author states that there can be no doubt of its superiority. To render innocuous the excretions of one man costs one farthing per day. Xeroform (tribromphenolbismuth), in the form of a powder, has been advocated by Hesse and Heusse[8] as a substitute for iodoform in non-infected wounds, excisions, &c., also for gonorrhœa. It is non-toxic, quite inodorous, has no irritating properties on the tissues or diseased mucous surfaces, and combines the therapeutic qualities of the phenol series with those of bismuth. Airol, the iodo-bismuth substitute for iodoform, continues to be extensively used for surgical purposes on the Continent. Besides its antiseptic, non-irritant, and other qualities previously noted in The Hospital, it is four times more bulky, and has therefore the advantage of being cheaper in use than iodoform.[9] Salol dissolved in liquid vaseline has been re-introduced by Dr. Colombini[10] as a local, non-irritating, and sufficiently powerful antiseptic. In the presence of alkaline fluids or living tissues salol appears to break up into salicylic and phenol in the nascent state. When split up in this way the author found that, while the good antiseptic action of each of the acids was maintained, their irritant action was absent, either from the way in which they were set free or from the small quantities of the acids evolved. Dr. Paul Thiéry[11] discusses the utility of picric acid as a local application in the treatment of conditions such as burns, where the deeper layers of the true skin have not been entirely destroyed. It is analgesic, antiseptic, and keratoplastic; it is neither caustic nor toxic, and may be used in the case of children. The best results are obtained if the dressing is applied as soon as possible after the burns, and if no other treatment has been previously adopted. A saturated solution made with distilled water is employed, and where the burn or abrasion is extensive it is recommended that the part should first be totally immersed in a bath of the fluid and then dressed with compresses of lint or gauze wrung out of the solution; these are covered with absorbent wool and bandaged on, so that the dressing may become dry. For burns of the first degree healing is often obtained in one or two days after the first application. In deeper burns it may be necessary to reapply the dressing once or twice. One inconvenience is that the dressings discolour the hands—india-rubber gloves should therefore be used. Professor E. Graser[12] presents the results of recent studies in the primary adhesion of wound surfaces by nature's collodion. It is probable, he says, that the adhesion of these surfaces, similar to that of endothelial surfaces, is due to a coagulation necrosis, in which the cells of the part give up the fibrin ferment necessary for the formation of the adhesion-reducing substance. Of course, the fibrin produced in this way is only a temporary adhesive material, and in a comparatively short time proliferation of fixed tissue cells in the neighbourhood makes the union a definite one. The writer in the *N.Y. Medical Record*[13] says, " we are safe in premising that aseptic surgery is carried to greater perfection in Germany than anywhere else in the world," and quotes from Mr. G. W. S. Farmer's admirable article in the *Medical Magazine* for March on "Aseptic Surgery in Germany." Mr. Farmer makes three divisions of his subject: (1) The preparation of the patient; (2) the preparation of surgeon and assistants; (3) the operation itself, and gives details for each. Everything turns upon absolute cleanliness before and during the operation rather than afterwards. It is asepsis pure and simple against antisepsis, always imperfect and always difficult; asepsis prevents infection, but antisepsis cannot effectually kill all the morbific germs in a wound already infected. The New York writer alluded to states that American surgeons are in all essential respects up to date, are firm endorsers of the German practice, and staunch believers in the principles of absolute cleanliness. Dr. G. E. Brewer[14] gives an interesting account of operative surgery at the New York City Hospital, and a report on the study of wound infection and its prevention at this institution; while Dr. E. R. Kirby[15] describes the surgical technique of Dr. J. W. White's clinics in the University of Pennsylvania under the following headings: (1) The preparatory treatment of the patient; (2) the operating room; (3) surgeon, assistants, and nurses; (4) instruments, ligatures, sutures, gauzes, sponges, dressings, &c.; (5) basins, trays, jars, &c. Each of these he treats in detail. The treatment of aseptic wounds without bandages or dressings, as carried out at Burnley, is presented by Dr. J. Mackenzie[16] as follows: (1) To maintain the parts at rest and the raw surfaces that are intended to unite together in close and constant apposition; (2) to prevent contamination of the wound from external sources; (3) to get rid of excessive discharges. The first is effected by the systematic employment of rows of buried catgut sutures, from the bottom of a wound to the skin edges, till finally the latter come together without tension. As to the second, a solution of celloidin is applied over the line of skin incision and for half an inch on either side. This solution quickly dries, leaving a thin transparent film that adheres to the skin with great tenacity, and yet is sufficiently plastic to adapt itself to the inequalities of the skin produced by movements of the parts. As regards the third point, when the wound is large the following method of drainage is employed. Before the wound is closed at the most dependent part, a small hole is made through the skin, and subjacent tissues as far away as possible from the wound. An unperforated piece of drainage tube larger than the hole in the skin is passed through into the wound, while its outer portion is introduced into a bag of gutta percha tissue containing a sponge soaked in strong antiseptic. The mouth of the bag is closely sealed to the tube. Dr. F. Page[17] tabulates the results of major amputations treated antiseptically in the Royal Infirmary, Newcastle-upon-Tyne, during the year 1895, and for a period of 17 years and 9 months. From April 1st, 1878, to December 31st, 1895, 938 major amputations were performed, and 80 patients died—8·5 per cent.; 365 were for injury, nearly all primary amputations, with 48 deaths—13·1 per cent.; 573 were for disease, and 32 patients died—5 5 per cent.

[1] Centralblatt für Gynäkologie, Feb. 29th, and N.Y Med. Jour., March 21, 1896. [2] Les Nouveaux Remèdes, Feb. 24, 1896. [3] Ibidem., p. 133. [4] Zeitschr. für Hygiène, April, 1896, and Revue des Nouveaux Remèdes, June 8, 1896, p. 371. [5] Therapeut. Monatshefte, Feb., 1896, and Epit. Brit. Med. Jour., June 6. 1896. [6] Wiener Med. Presse, March 29, and Epit. B.M. Jour., June 6, 1896. [7] The Medical Week, Feb 14, 1896. [8] Ther. Monatsh., April, 1896, and Les Nouveaux Remèdes, May 24, 1896, p. 295. [9] Pamphlet, R. W. Greeff and Co., London, July, 1896. [10] Med. Record, Feb 15, 1896, and Riforma Medica. [11] Gazette des Hôpitaux, Jan. 18 and Feb. 27, and Practitioner, June, 1896, and Internat. Med. Mag., May, 1896. [12] Indian Med. Re., April, 1896, and Archiv. f. Klin. Chirurgie. [13] N.Y. Med. Record, April 11, 1896, p. 521 [14] N.Y. Med. Jour., May 2, 1896, p. 569. [15] Therapeutic Gazette, May 15, 1896, p. 315. [16] Brit. Med. Jour., Feb. 1, 1896, p. 267. [17] Lancet, March 21, 1896, p. 763.

